# Advances in attractive therapeutic approach for macrophage activation syndrome in COVID-19

**DOI:** 10.3389/fimmu.2023.1200289

**Published:** 2023-07-06

**Authors:** Shunyao Chen, Cong Zhang, Deng Chen, Liming Dong, Teding Chang, Zhao-Hui Tang

**Affiliations:** ^1^ Department of Trauma Surgery, Emergency Surgery & Surgical Critical, Tongji Trauma Center, Wuhan, China; ^2^ Department of Emergency and Critical Care Medicine, Tongji Hospital, Tongji Medical College, Huazhong University of Science and Technology, Wuhan, China

**Keywords:** macrophage activation syndrome (MAS), COVID-19, SARS-CoV-2, therapy, cytokine storm syndrome (CSS)s

## Abstract

Nowadays, people have relaxed their vigilance against COVID-19 due to its declining infection numbers and attenuated virulence. However, COVID-19 still needs to be concern due to its emerging variants, the relaxation of restrictions as well as breakthrough infections. During the period of the COVID-19 infection, the imbalanced and hyper-responsive immune system plays a critical role in its pathogenesis. Macrophage Activation Syndrome (MAS) is a fatal complication of immune system disease, which is caused by the excessive activation and proliferation of macrophages and cytotoxic T cells (CTL). COVID-19-related hyperinflammation shares common clinical features with the above MAS symptoms, such as hypercytokinemia, hyperferritinemia, and coagulopathy. In MAS, immune exhaustion or defective anti-viral responses leads to the inadequate cytolytic capacity of CTL which contributes to prolonged interaction between CTL, APCs and macrophages. It is possible that the same process also occurred in COVID-19 patients, and further led to a cytokine storm confined to the lungs. It is associated with the poor prognosis of severe patients such as multiple organ failure and even death. The main difference of cytokine storm is that in COVID-19 pneumonia is mainly the specific damage of the lung, while in MAS is easy to develop into a systemic. The attractive therapeutic approach to prevent MAS in COVID-19 mainly includes antiviral, antibiotics, convalescent plasma (CP) therapy and hemadsorption, extensive immunosuppressive agents, and cytokine-targeted therapies. Here, we discuss the role of the therapeutic approaches mentioned above in the two diseases. And we found that the treatment effect of the same therapeutic approach is different.

## 1 Introduction

Since December 2019, a new β-coronavirus named severe acute respiratory syndrome coronavirus 2 (SARS-CoV-2) has led to an outbreak around the world, which has posed a global challenge (1–3). According to the World Health Organization (WHO) Dashboard (https://covid19.who.int/), up to 24 May 2023, there have been 766,895,075 confirmed cases of COVID-19, including 6,935,889 deaths. Even now that the number of COVID-19 vaccinations has reached 13355264024, there are still two currently circulating variants of interest (VOI), XBB.1.5 and XBB.1.16,and seven currently circulating variants under monitoring (VUMs) (Tracking SARS-CoV-2 variants (who.int)). These variants have been associated with an increase in the transmission or mortality of COVID-19 (4–9), or may escape immunity when compared to the original strain or D614G variant (9–14). Despite the current trend of COVID-19 being in decline, the harm of COVID-19 cannot be underestimated due to the great possibility of its resurgence.

The imbalanced and hyper-responsive immune system plays a critical role in COVID-19 pathogenesis. The immune response of SARS-CoV-2 infection is characterized by the differentiation and proliferation of various types of immune cells and the release of immune mediators (15, 16). Early evidence suggests that the clinical and laboratory features of the patients severely infected with COVID-19 are similar to the clinical phenotypes of “Cytokine Storm Syndromes” (CSS) (17–19).In general, the patterns and levels of inflammation and immune dysregulation observed in the periphery and lungs of COVID-19 patients are characteristic of “Cytokine Storm Syndromes”. Acute respiratory distress syndrome (ARDS) and Multiple organ dysfunction syndrome (MODS) are the main causes of mortality in patients with COVID-19, while CSS will lead to ARDS and MODS (20–25). Therefore, CSS plays a very critical role in patients with severe COVID-19.

CSS refers to a diverse set of conditions that collectively manifest a clinical phenotype of hyperinflammation, hyperferritinemia, and multiorgan failure. The spectrum of cytokine storm syndromes spans wide-ranging conditions, one of which is called Macrophage Activation Syndrome (MAS) (26). MAS is a prototypic form of CSS that develops in many rheumatic diseases, such as the Still’s disease spectrum (systemic juvenile idiopathic arthritis [sJIA] and adult-onset Still’s disease) (27), systemic lupus erythematosus (28) and Kawasaki disease (29). Considerable studies have been performed to elucidate that MAS is involved in COVID-19 associated with worse disease severity and poorer prognosis (30). Overzealous immune responses associated with MAS, may be driving COVID-19 related ARDS (21). There has been research shows that treatment of MAS, such as the use of tocilizumab and the combination of tocilizumab and steroids, significantly reduced the intubation or death of severe patients (31, 32).

While the new case of COVID-19 infections is decreasing, it is premature for ceasing the research on its treatment when there is so much uncertainty about how SARS-CoV-2 will behave, the path of its mutations, as well as its many possible long-term effects. Standard treatment for MAS includes several immunosuppressive drugs (33). Furthermore, many cytokines targeted therapeutic agents have achieved good results in the treatment of MAS, such as Anakinra, Tocilizumab, etc. al (30, 34–36). However, further research into SARS-CoV-2 pathogenesis has found some differences between MAS and COVID-19, which makes the therapeutic effect uncertain. More recently, randomized trials of anti-IL-1 and IL-6 agents have found little evidence of overall benefit (37–39). It is obvious that the effect of therapeutic interventions for MAS in COVID-19 is controversial. To address this issue, in this review, we systematically summarize the advances in attractive therapeutic approaches to treat MAS for COVID-19.

## 2 The MAS of COVID-19

MAS is a fatal complication of immune system disease, which is caused by the excessive activation and proliferation of macrophages and cytotoxic T cells (CTL). MAS can lead to a cytokine storm and a persistent inflammatory state (40, 41). This disease is caused by the combination of the following three conditions (1): the inability of the cytolytic function of NK cells and CTL cells (2); the high sensitivity of macrophages caused by the high response of macrophage pattern recognition receptors (PRR) to pathogen-associated molecular patterns (PAMP) and the underlying inflammatory symptoms of the body;(3) trigger factors (42). Under the above conditions, the end product is escalating production of cytokines. The main diagnostic criteria that contribute to the development of a diagnostic score for MAS include: Fever, hepatosplenomegaly, hyperferritinemia, hepatopathy, coagulopathy, thrombocytopenia, hypertriglyceridemia, decrease in erythrocyte sedimentation rate and bone marrow hemophagocytosis (30, 43).

COVID-19 related hyperinflammation shares common clinical features with the above MAS symptoms, such as hypercytokinemia, hyperferritinemia, coagulopathy (2, 18, 19, 21, 44) (17). Some scholars proposed some classification framework for identifying COVID-19 cytokine storms according to diagnostic and classification criteria of MAS, such as cHIS (COVID-19 associated hyperinflammatory syndrome) and COVID hyperinflammation (COV-HI) (45, 46).

**Figure 1:**
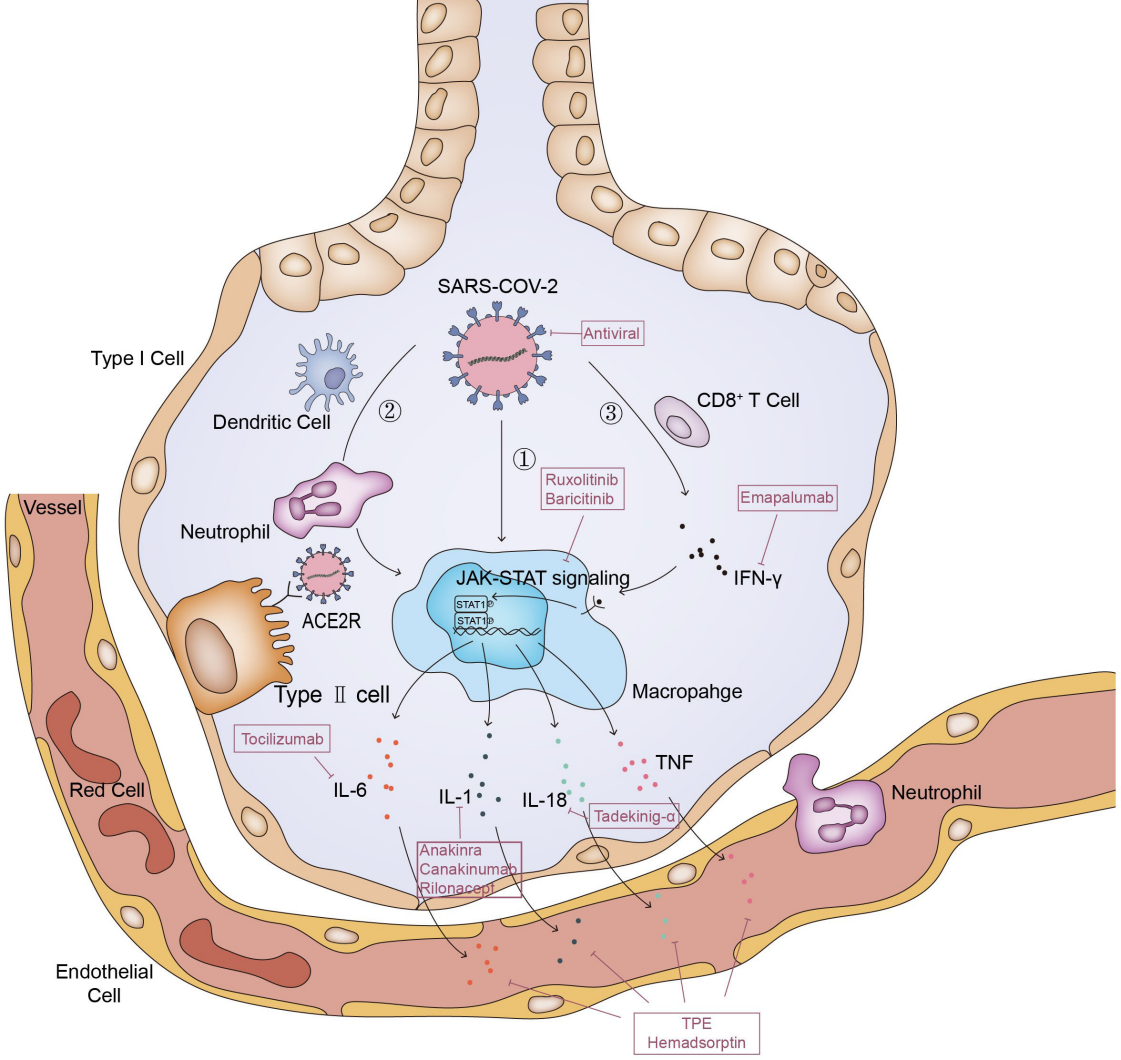
Pathways of MAS in COVID-19. The entry of SARS-CoV-2 into cells depends on the binding of S protein on the surface of virus particles to the ACE2 receptor of cells; **①**. Macrophage ingests viral RNA through phagocytosis, phagocytosis of alveolar epithelial cells infected by SARS-CoV-2 viruses or phagocytosis of viruses through Fc receptor mediated endocytosis. This leads to abnormal activation of macrophages to release cytokines; **②**. Dendritic cells and neutrophils will also be recruited here. On the one hand, neutrophils and dendritic cells will produce a variety of cytokines under the stimulation of SARS-CoV-2, on the other hand, they will also transmit activation signals to macrophages, making them release cytokines and chemokines; **③**. SARS-CoV-2 stimulates anti-viral immune pathways with CD8^+^ T cell expansion. Expanded CD8^+^ T cells produce interferon-γ (IFN-γ), IFN-γ binds the IFN-γ receptor and further stimulate macrophage activation to produce multiple anti-inflammatory cytokines through the JAK-STAT pathway. And some of these cytokines signal *via* the JAK-STAT pathway, including IL-6 and IFN (but not IL-1 and TNF), to regulate cell homeostasis, proliferation and differentiation as well as control the immune system and inflammatory response. Inadequate cytolytic capacity due to immune exhaustion and/or defective antiviral responses leads to prolonged cross-talk between CD8^+^ T cell, Antigen-presenting cells (APCs) and alveolar macrophages, contributing to the further release of many proinflammatory cytokines.

The entry of SARS-CoV-2 into cells depends on the binding of S protein on the surface of virus particles to the ACE2 receptor of cells, and the activation of S protein by the host membrane serine protease TMPRSS2 (47, 48). SARS CoV-2 virus was found in alveolar macrophages in autopsy of SARS CoV-2 pneumonia patients, but the macrophages do not express ACE2 and TMPRSS2 (49). Therefore, the entrance of virus into macrophages should be achieved by other means (35, 50).This leads to abnormal activation of macrophages to release cytokines including IL-6, which is one of the driving factors of MAS (51–53). In MAS, immune exhaustion or defective anti-viral responses leads to inadequate cytolytic capacity of CTL which contributes to prolonged interaction between CTL, APCs and macrophages (54). It is possible that the same process also occurred in COVID-19 patients, and further led to a cytokine storm confined to the lungs (55)(**Figure 1**).In deed, in addition to macrophages, there are many other cells, which will release a large number of cytokines and chemokines, thus leading to the inflammatory cascade reaction to further expand the cytokine storm (56).And then, the local inflammation induced by SARS-CoV-2 infection spread rapidly to the entire lung (57). Accumulation of immune cells accelerates the progression of lung inflammation into ARDS, which is associated with the poor prognosis of severe patients such as multiple organ failure and even death (24, 35).

**Figure 2:**
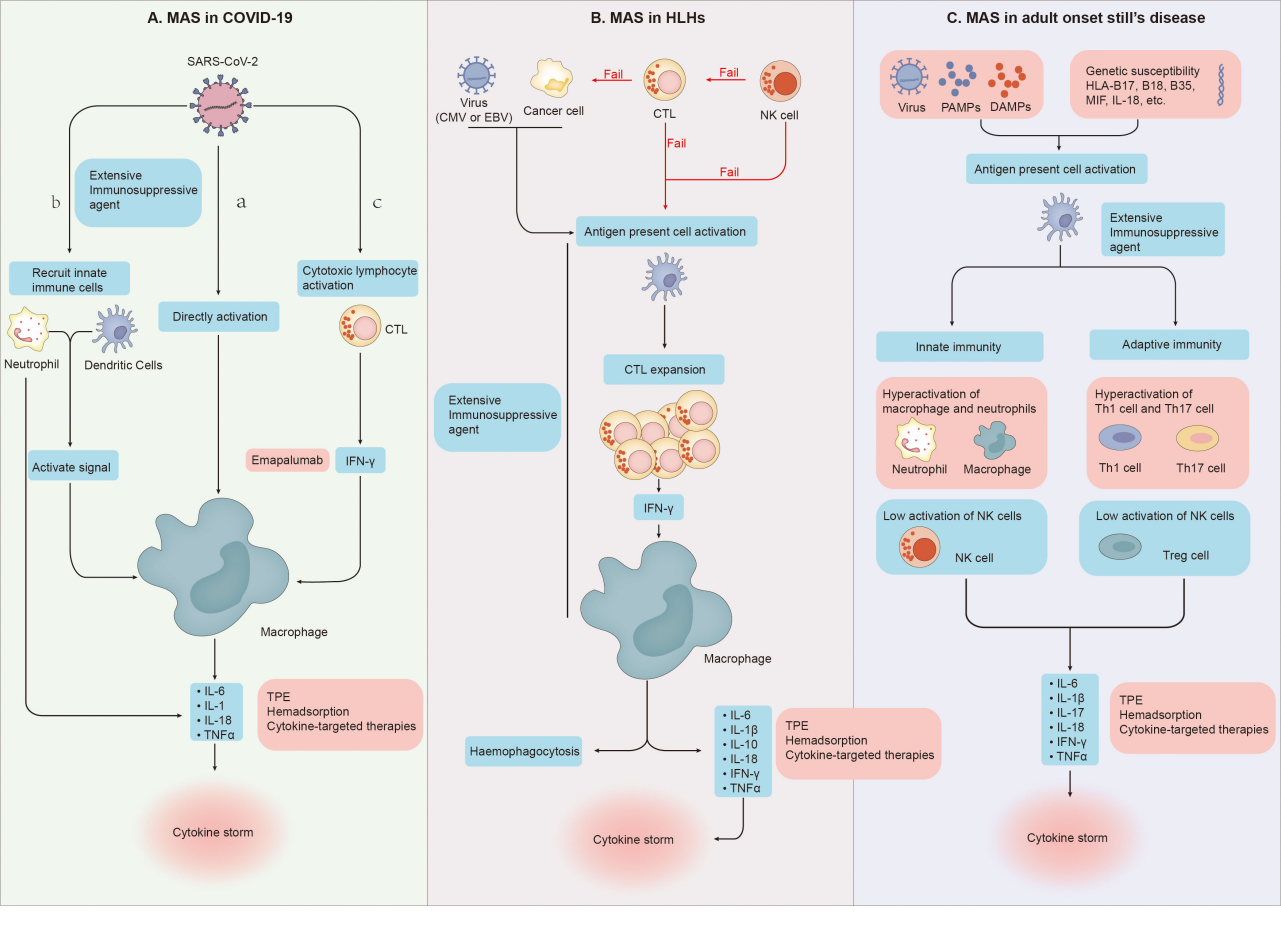
**(A) (a)** SARS-CoV-2 directly leads to abnormal activation of macrophages to release cytokines. **(b)** Dendritic cells and neutrophils will also be recruited. On the one hand, neutrophils and dendritic cells will produce a variety of cytokines under the stimulation of SARS-CoV-2, on the other hand, they will also transmit activation signals to macrophages, making them release cytokines and chemokines. **(c)** SARS-CoV-2 stimulates anti-viral immune pathways with CTL expansion. Expanded CTL produce interferon-γ(IFN-γ), IFN-γ binds the IFN-γ receptor and further stimulate macrophage activation to produce multiple anti-inflammatory cytokines. **(B)** Cytotoxic function of NK cells fails to clear tumor or infected cells and cytotoxic T cells. Cytotoxic function of CTLs fail to clear tumor cells and APCs. And then Proliferation of the population of activated CTLs induce activation and proliferation of tissue macrophages (histiocytes). Further haemophagocytosis and cytokine storms occur. **(C)** Under the dual effects of non-infectious factors, bacterial or viral attacks, and the patient’s own genetic susceptibility, both the adaptive and innate immune systems are activated. In the innate immune system, the activity of macrophages and neutrophils is abnormally elevated, and the activity of NK cells is impaired. In the adaptive immune system, Th1 and Th17 activities increase while Treg activity decreases. Under the combined action of these immune cells, the body produces cytokine storms.

Although the process of macrophage over activation is very similar to that of MAS, there are still many differences between them, such as clinical manifestations, laboratory tests and specific forms of cytokine storm (2, 58, 59). In severe COVID-19, many studies have found the expansion of alveolar macrophages and the obvious increase of pulmonary inflammatory factors (IL-6, IL-8, IL-1β) (60–62). And David A. Dorward et al. have observed that although SARS-CoV-2 RNA exists in many organs, such as the gastrointestinal tract, liver and kidney, these extrapulmonary sites with evidence of viral transcription, did not have substantial local inflammation (63).The evidence above supports a model of a lung-centric, self-sustaining inflammatory loop leading to cytokine storm (55). However, in MAS, cell mediated cytotoxicity fails due to various reasons such as genetic or viral infection, which leads to the prolonged cell-to-cell interactions, the cascade expansion of proinflammatory cytokines, and then the generation of systemic cytokine storms (**Figure 2**). Cytokine storm will further lead to systemic macrophage activation, hemophagocytosis and multiple organ dysfunction (54). To sum up, it is easy to see that in terms of the specific form of cytokines storm, the main differences between them is the cytokines storm in COVID-19 pneumonia is mainly the specific damage of the lung, while in MAS is easy to develop into systemic (58, 59).

Precisely because of the similarity between over-activated alveolar macrophages in COVID-19 and MAS, the therapeutic approach of MAS might be able to analogize to COVID-19 patients, but the effect is still not so clear (55). Although some scholars believe that the concept of MAS in COVID-19 is relatively abstract (64), and many of the supporting evidence for drugs and treatments we have found are only case reports, with relatively limited reference value. However, the treatment of MAS like inflammation in COVID-19 could be improved by summarizing some treatment targets and interventions of MAS and we may be able to propose some potential treatment methods, so as to improve the prognosis of COVID-19.

## 3 The attractive therapeutic approach to prevent MAS in COVID-19

### 3.1 Early identification and active interventions

Early active intervention is necessary to improve the prognosis of MAS in COVID-19 patients, so early identification of MAS in COVID-19 patients is very important. However, in the research of Caricchio and colleagues, we found that some patients suspected of COVID-19 cytokine storm rarely met the diagnostic criteria of HLH/MAS, whether HLH-2004 or 2016 MAS Criteria (55, 65). Even HScore, a score recommended by Puja Mehta and colleagues to detect the inflammatory states of COVID-19 patients (21), was considered to lack sensitivity in managing hyperinflammatory states in patients by David L Leverenz in May 2020 and therefore not recommended for use (66).Therefore, some authors have developed new methods to identified the MAS in COVID-19. Webb and colleagues developed a classification framework called cHIS (COVID-19 associated hyperinflammatory syndrome) with 6 core features of CSS including: fever, macrophage activation, liver inflammation, hematologic dysfunction, coagulopathy, and hypercytokinemia (45). Besides, Manson et al. classified patients as having COVID hyperinflammation (COV-HI) with ferritin>1500 mg/L, or CRP >15 mg/dL or doubling in 24h (46). These scores and classifications have a very significant relationship with the prognosis of patients, and can identify the time for intervention earlier. It has also been applied in subsequent studies and clinical practice (67–69).

#### 3.1.1 Antiviral

Antiviral drugs have been widely concerned since the beginning of the epidemic. Remdesivir, a nucleotide analog, is the first direct antiviral drug. According to the data, early use of this drugs after diagnosis can reduce the symptoms and hospitalization rate of patients (70). However, the drug is expensive and must be injected intravenously (71–73). Recently, oral antiviral drugs for COVID-19 have achieved great progress. The United States (US) Food and Drug Administration (FDA) issued an EUA for the use of the molnupiravir and nirmatrelvir-ritonavir (Paxlovid) (74). In a recent meta-analysis, the existing data showed that these new oral antiviral drugs can effectively reduce the mortality and hospitalization rate of patients with COVID-19, and will not increase the incidence of adverse events (75). As mentioned earlier, MAS often occurs in patients with systemic inflammatory reaction in the late stage of infection, so the early application of these antiviral drugs may also have a very expectant effect on the prevention of MAS.

#### 3.1.2 Convalescent plasma (CP) therapy

Antibodies in convalescent plasma can kill or prevent further replication of the virus (76), and then further inhibit the inflammatory reaction and reduce the risk of excessive immune response (77). It has been proved that early administration of CP (within 9 days after the onset of symptoms) can significantly reduce the risk of hospitalization due to disease progression (78, 79). In a systematic review and meta-analysis of CP, there was a clear association between the use of CP and the mortality benefit of hospitalized patients with COVID-19 (80). In addition, inflammatory markers such as IL-6 and CRP are also less expressed in COVID-19 patients receiving CP blood transfusion (81). And IL-6 plays a key role in the pathogenesis of MAS (82). Therefore, the early use of CP may have active effect on the prevention of MAS in COVID-19.

However, the application value of CP in patients who require invasive mechanical ventilation is limited (83). Moreover, there are currently some issues with the collection of CCPs. Because most blood services do not actively collect CP, people can only use old stocks of CCPs. The effect of the old CP on the current epidemic strains may be limited, due to differences between variants of COVID-19 (84). It is impractical to conduct new experiments every time a new strain of virus appears, and collecting CCP again, so the application prospects of this treatment method are still relatively limited.

### 3.2 Therapeutic plasma exchange (TPE) and hemadsorption

Although the treatment of the virus itself is certainly desired, hypercytokinemia will occur in COVID-19 associated hyperinflammation, which will lead to sever cytokine storm. Therefore, it is also a necessary treatment to eliminate inflammatory cytokines through Therapeutic Plasma exchange (TPE) and Hemadsorption.

With the background therapy of glucocorticoids and immunosuppressive agents, Plasma exchange is an effective approach to rapidly clear inflammatory cytokines and reduce mortality of autoimmune inflammatory rheumatic diseases (AIIRD)-associated MAS (85). A prospective randomized controlled trial of Yuan YH et al. showed that incorporation of plasma exchange could reduce the levels of CRP, procalcitonin, alanine transaminase and total bilirubin, reduce the stay time in ICU and increase the response rate to other treatments (86). Satoshi Sato et al. also reported a case recently, after using standard treatment and immunosuppressions, the patient still did not achieve clinical remission. However, after initiation of PE, the patient’s symptoms improved significantly (87). The evidences above provide a practical basis for applying this method to COVID-19.

As early as 2020, Philip Keith et al. have proposed the possibility of applying TPE to fulminant COVID-19 (88). In fact, a lot of evidence has emerged to prove that TPE can significantly reduce the mortality rates of COVID-19 since then (89–97). In a recent meta-analysis conducted by JinlvQin et al., it was proposed that TPE significantly reduced the mortality of hospitalized patients with moderate risk COVID-19 (98).What is more remarkable is that TPE can significantly reduce the levels of IL-6, ferritin and CRP in COVID-19 in these studies. As mentioned earlier, these laboratory indicators are markers of MAS. Therefore, we have reason to believe that TPE has therapeutic and even preventive effects on MAS, and has obvious benefits for the prognosis of patients.

**Figure 3:**
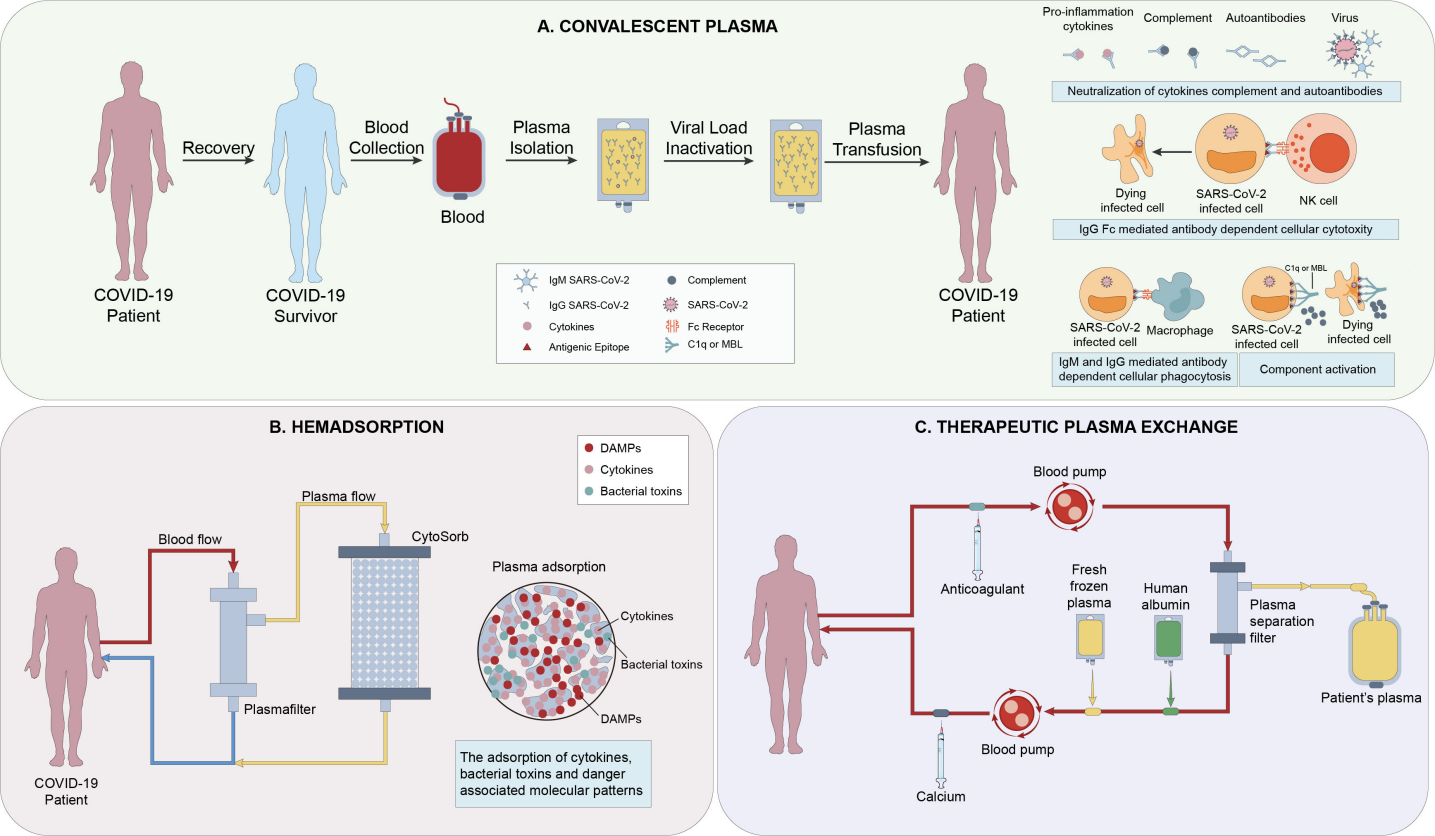
**(A)** The mechanisms of action of Convalescent Plasma(CP) include direct antiviral effects (eg, viral neutralization), viral clearance *via* immunoglobulin (Ig M and IgA-mediated neutralization, non-neutralizing IgG Fc-mediated functions (eg, antibody-dependent cellular cytotoxicity, phagocytosis and complement activation), and immunomodulation (1). **(B)** Hemadsorption is a technique in which a sorbent is placed in direct contact with blood in an extracorporeal circuit. CytoSorb is a highly adsorptive and biocompatible polymer able to eliminate various mediators (e.g., IL-1/6/8/10), bacterial toxins, and danger-associated molecular patterns (DAMPS) from the bloodstream (2). **(C)** Plasma exchange involves separating and discarding the plasma and blood cells in a patient’s body, and replacing them with an equal amount of fresh frozen plasma or human albumin exchange solution. There are metabolic toxins and various inflammatory mediators of all sizes present in the discarded plasma (3).

Hemadsorption can also significantly reduce the high level of cytokines in the patient’s circulation (99). But unlike PE, hemadsorption does not require plasma separation and allows for simultaneous fluid removal (100)(**Figure 3**). Recently, many reports have found that this treatment can rapidly reduce the level of IL-6 and IL-10, significantly improve hemodynamics, and without relevant associated adverse effects (101, 102). Therefore, hemadsorption can be used as an effective and safe rescue treatment for patients with MAS and multiple organ dysfunctions, and complementary to standard protocol treatments (103, 104). In a randomized controlled trial, it was clearly proved that Hemadsorption can early relieve organ dysfunction in critically ill patients with COVID-19 and stabilize the clinical symptoms, although it has no significant impact on inflammatory indicators and 28 day mortality (105). Besides, in a case report by Rajib Paul et al, they proposed that Hemadsorption was effective in providing hemodynamic stability, improving organ dysfunction, and modulating the cytokine storm (106).

To sum up, TPE has therapeutic and even preventive effects on MAS of COVID-19 patients, and can reduce the mortality of patients. But hemadsorption may not have a direct impact on the mortality, but it can also stabilize the patient’s general condition to provide time for further treatment.

### 3.3 Extensive immunosuppressive agent

#### 3.3.1 Steroids

According to the Consensus-Based Guidelines for the Recognition, Diagnosis, and Management of Hemophagocytic Lymphohistiocytosis in Critically Ill Children and Adults, corticosteroids are the backbone of the therapy of MAS (107). Moreover, the efficacy of corticosteroids as a basic treatment for MAS has been proven in many studies and clinical practices (108–110). Sathish Loganathan et al. reported two cases using tapering dose regimen of intravenous methylprednisolone (IVMP), and they achieved good results (111). In addition, early treatment of steroids can help stabilize the disease and buy time for further diagnosis and treatment (107).

But in the initial expert opinion, steroids are discouraged for use in viral lung infections, including COVID-19 (112). They believe that the immunosuppressive effect of steroids will lead to prolonged viremia and increased risk of bacterial coinfection. And based on some past research experience in the treatment of SARS-CoV-1 (113) and Middle East respiratory syndrome (MERS)-CoV (114), they believe that the use of steroid drugs should be very cautious.

However, in February 2021, Peter Horby et al. reported for the first time that dexamethasone (6 mg once daily for up to 10 days) reduced the 28-day mortality of hospitalized patients with COVID-19 through a randomized controlled trial (115). In a previous prospective meta-analysis of clinical trials in 257 critically ill patients with COVID-19, the use of systemic corticosteroids was associated with lower 28-day 258 all-cause mortality (116). In a recent meta-analysis conducted by Manisha Thakur et al., it further explained that the steroids play a significant role in the decrease of demises of hospitalized COVID-19 patients (117).

Frank L. van de Veerdunk et al. believes that the observation that the beneficial effects of steroids are significant in sicker patients could be explained by the pleiotropic effects of steroids (118). However, they also suggested that excessive use of corticosteroids, especially in the early stage of disease, may be harmful. In addition, some experts suggested that glucocorticoids should be used, but only at the right time (119). In other words, glucocorticoid can only be prescribed in the inflammatory phase of COVID-19 (120, 121). This is different from the therapeutic backbone role of steroids in MAS. Steroid is a systemic and widespread immunosuppressive drug, so it is easier to benefit from the treatment of MAS, while the use of early COVID-19 patients should be cautious, and it is easier to benefit from severe COVID-19 patients.

#### 3.3.2 Calcineurin inhibitors (CNIs)

The two most common CNIs drugs are cyclosporine A (Cys) and tacrolimus (TAC), which are most commonly used in solid organ transplantation(SOT) (122) and systemic rheumatic diseases (123). When MAS develops to moderate or response to steroids is not evident, early initiation of cyclosporine a rapid and effective treatment option (124–126). Besides, later studies showed that anti thymocyte globulin (ATG) may be a safer choice for patients who failed to respond to the combined treatment of steroids and cyclosporine A (127–129).

As early as July 2020, Ren é Hage et al. found that a few of the patients with SOT who used tacrolimus reported severe COVID-19, and proposed that this type of drugs might prevent or even treat the excessive inflammation caused by SARS CoV-2 infection (130). This therapeutic drug may be superior to steroids. On the one hand, cyclosporine A can protect the upstream of cytokines in patients with COVID-19, rather than just targeting proinflammatory cytokines (131).On another hand, Long term and chronic use of glucocorticoids may lead to severe COVID-19, complications, etc., while chronic CNIs treatment has no effect on mortality (132). In addition to its immunosuppressive effect, CNIs can also prevent the occurrence of MAS from the upstream of cytokine storm through its antiviral activity shown in many RNA viruses (133, 134) and the ability to prevent mitochondrial failure (135, 136).

However, like the application of other extensive immunosuppressive drugs in viral infections, CNIs may lead to uncontrolled initial viral replication, viral immune evasion, and higher mortality rates (137–139). Moreover, there is still a lack of prospective and randomized clinical trials to demonstrate the specific effects of CNIs in the treatment of COVID-19.

### 3.4 Etoposide

The etoposide (VP-16)-based regimens, a kind of treatment approach for MAS, include HLH-94, HLH-04 and the modified Doxorubicin−Etoposide-Methylprednisolone (DEP) regimen (140).

In the HLH-94 and HLH-2004 regimens, the incorporation of etoposide and dexamethasone has been proposed as the treatment pillar. Karin Palmblad et al. found that the use of etoposide can lead to clinical improvement coinciding with a decline of systemic HMGB1, IL-18, IFN-γ, and ferritin levels (141). Although there is some evidence that this drug may aggravate the infection or make it easier to develop new infections, its benefits are obviously greater (140).

Modified Doxorubicin−Etoposide−Methylprednisolone (DEP) regimen has attracted much attention since it was proposed. Doxorubicin can quickly eliminate the overactivated macrophages and T cells (142). Besides, the dose of the glucocorticoid in the regimen was reduced to avoid the adverse effects in patients who failed to achieve remission after glucocorticoid pulse therapy. Recently, the research hold by He, L. et al. have demonstrated that modified DEP regimen is a promising alternative therapy for adults with R/R MAS owing to the high response rate, rapid action, and satisfactory tolerance (143).

As early as 2020, some people put forward the idea of using etoposide to treat severe patients with COVID-19 accompanied by systemic inflammation (144–146). In a mouse model study, the combination of low-dose etoposide and prednisolone improved the survival rate of fatal ARDS model mice, which all suffered from hypercytokinemia and MAS (147). They also found that this treatment combination can inhibit the recruitment and activation of macrophages, T cells, NK cells and neutrophils in the lung. And it has been proved that early administration of etoposide is a treatment option for EBV-related MAS patients (148). So similarly, low-dose etoposide may play a therapeutic role in treating MAS in COVID-19. Akiyoshi Takami believes that low-dose etoposide can update the CTL that has lost its function so that the abnormally activated macrophages and SARS-CoV-2 infected cells are eliminated, thus recovering the abnormal immune regulation associated with SARS-CoV-2 infection (146). A clinical trial lasting from May 8, 2020 to the present will likely contribute to further evaluate the safety and efficacy of etoposide in COVID-19 patients (ClinicalTrials.gov Identifier: NCT04356690).

### 3.5 Cytokine-targeted therapies

As mentioned earlier, many cytokines play a key role in the MAS caused by individual MAS and COVID-19, so the treatment methods targeting these cytokines are also the focus of current research.

#### 3.5.1 IL-1

IL-1 blockers mainly include anakinra, canakinumab, rilonacept, gevokizumab and bermekimab.

Enough clinical trials have proved that anakinra can achieve obvious benefit in the treatment of macrophage activation syndrome (MAS), sepsis with MAS, and severe acute respiratory syndrome coronavirus 2–associated cytokine storm (149, 150).Recently, a retrospective analysis of 218 COVID-19 patients with MAS conducted by S. Amikishiyev et al. suggests that starting anakinra earlier in hospitalized patients may provide better results (151). Another retrospective analysis involving 16 patients showed that the patients had good tolerance to anakinra compared with the above etoposide-based therapy anakinra (152).Besides, there is a case report and a single-center, retrospective experience highlight that anakinra use in treating MAS is effective (153, 154). Therefore, there is no doubt that Anakinra has a positive effect on the treatment of MAS. In fact, this drug has also been included in the Consensus-Based Guidelines for the Recognition, Diagnosis, and Management of Hemophagocytic Lymphohistiocytosis in Critically Ill Children and Adults (107).

However, anakinra’s treatment effect on patients with COVID-19 is still controversial so far. A cohort study recorded a clear benefit from the use of this drug in severe forms of COVID-19 (155). They put forward that anakinra reduced both need for invasive mechanical ventilation in the ICU and mortality among patients with severe forms of COVID-19, without serious side-effects. Similarly, this view was put forward by two other cohort studies in the same period (156, 157). However, in a later randomized controlled experiment, totally different results were obtained (38). The experimental results showed that Anakinra could not effectively reduce the need for mechanical ventilation and mortality in patients with mild to moderate COVID-19. So, up to now, there are still several related studies on this drug in progress (ClinicalTrials.gov Identifier: NCT04357366 NCT04362111). Elnaz Khani et al. summarized the existing evidence on the use of Anakinra in COVID-19 (158). They believed that the benefits of Anakinra in patients with high inflammation were highly related to the duration of treatment, drug dose, route and time of administration. This is very similar to the treatment of anakinra in MAS. In the early stage of inflammation, around the first week of symptom onset, when patients need to supplement oxygen and do not undergo invasive mechanical ventilation within about 10 days, high dose of anakinra (>100 mg) may be effective and improve the results.

In addition to anakinra, canakinumab are also being tried for MAS treatment. Some studies have shown that canakinumab administered at doses of 4 mg/kg monthly does not have a major effect on the risk of developing MAS or on its clinical features (159). Some scholars believe that this is related to the dose. The monthly dose does not exceed 4 mg/kg, which may not be sufficient to neutralize the excessive IL- 1β in MAS activity. Kostik et al. recently described eight patients with sJIA treated with canakinumab. Several patients had the dose of canakinumab increased and they experienced the resolution of MAS. And there were no notable short-term adverse events (160). In a case-based review of Deniz Gezgin Yıldırım et al., a case that did not respond to anakinra but well responded to canakinumab was reported (161). Although this drug is more used in sJIA patients. But this may provide a new possibility for the management of MAS in COVID-19, especially for patients who do not respond to anakinra.

In August 2020, Ucciferri et al. described for the first time through a retrospective analysis that subcutaneous injection of canakinumab 300mg can rapidly reduce systemic inflammatory reaction and improve the oxygenation of COVID-19 patients (162). Lorenza Landi et al. also proved that this drug can reduce the inflammatory markers of patients with COVID-19 and improve the survival rate of patients (163). Furthermore, Guangyu Ao et al. infer that canakinumab could mitigate and even prevent immune-mediated tissue damage and organ dysfunction by limiting the level of acute inflammation and propensity for the activation of a cytokine storm (164–166). However, some people believe that the treatment of severe COVID-19 with canakinumab may increase the incidence rate of severe infection (167). And severe infection will induce macrophage activation syndrome. So, more trials are needed to prove the safety and efficacy of canakinumab injection in prevent MAS in COVID-19.

Besides, rilonacept is approved for the treatment of IL-1-mediated diseases too. Although rilonacept can improve some inflammatory diseases such as sJIA (168), familial cold autoinflammatory syndrome (FCAS) and Muckle-Wells syndrome (MWS) (169). However, rilonacept does not have much evidence and research to prove its therapeutic effect in COVID-19, just as it is in the treatment of MAS.

Overall, Anakinra seems to be superior to the other two drugs in the actual treatment process, which may be due to its better safty, rapid effect, short half-life and its ability to block both IL-1α and IL-1β. The increased dosage of canakinumab may provide a new possibility for the treatment of patients who do not respond to anakinra. The treatment of Rilonacept in MAS is relatively rare. It is easy to find that the effect of cytokines targeting drugs against IL-1 in preventing MAS in COVID-19 is different from that in patients with MAS alone, which may be related to the difference between MAS and MAS in COVID-19 mentioned above. Therefore, more research is needed to ensure the efficacy of these drugs.

#### 3.5.2 IL-6

Tocilizumab is a recombinant humanized IL-6 receptor monoclonal antibody. Although it has been reported previously that tocilizumab can reduce the clinical symptoms of MAS (170).A recent single-center observational study found that up to 6 of the 20 patients with adult-onset Still’s disease (AOSD) under tocilizumab treatment developed clinically diagnosed MAS (171). In a case report, a patient successfully treated AOSD with tocilizumab at the beginning, but when it relapsed, it quickly developed symptoms of MAS after using tocilizumab again (172).However, in another case report, the combination of anakinra and tocilizumab successfully produced significant clinical improvement in a critically unwell patient (173). This may be because the selective inhibition of IL-6 by tocilizumab alone enhances the production of other proinflammatory cytokines such as TNF-α, IL-1, IL-18, etc. in AOSD patients prone to MAS (172).

In view of the efficacy of tocilizumab in MAS and the key role of IL-6 in COVID-19, Bingwen Liu et al. proposed to use this drug for COVID-19-induced CRS (20). This view has been gradually confirmed and improved. In a recent systematic review of a randomized clinical trial, Driton Vela et al. proposed that the use of tocilizumab in patients with moderate and severe COVID-19 could reduce all-cause mortality without increasing the number of serious adverse events (174). In another recent systematic review, they believed that immunosuppressants can significantly reduce mortality and have no effect on the increase of the risk of secondary infection (175). In terms of secondary infection, an umbrella review in August 2022 also put forward same views (176, 177). Their research shows that the treatment of steroids or steroids plus tocilizumab did not confer a higher risk of bacterial infections and improved survival rates. As mentioned above, overlapping infection is one of the inducements of MAS (42). Tocilizumab will not increase the risk of overlapping infection when reducing the inflammatory indicators of patients and blocking the cytokine storm, so it has an optimistic prospect for the management of MAS in patients with COVID-19.

However, the population and time of application of this drug are very critical. In a randomized controlled trial, they found that among patients with severe or critical COVID-19, tocilizumab did not benefit, and even increased mortality (178). The reason for this phenomenon was later explained in a review, in the early stage of COVID-19, cytokines may have a protective effect, while in the late stage, IL-6 will be cis phagocytic regulation, promoting cell growth and survival (179). Therefore, intervention measures should be taken in the second week of symptom onset (or within 7 days after hospitalization) to be benefit to patients, which could only disrupt the presentation and trans-signaling of pro-inflammatory IL-6 (180).

Therefore, the efficacy and safety of IL-6 monoclonal antibodies in the treatment of MAS in COVID-19 and in the treatment of COVID-19 itself need to be more fully evaluated through further prospective and well-designed clinical studies with larger sample sizes and long-term follow-up.

#### 3.5.3 IL-18

There is evidence that the level of IL-18 in COVID-19 patients with MAS and ARDS is significantly higher than that in patients without MAS and ARDS, and high levels of IL-18 and IL-1Ra also seem to be related to the mortality of patients (181).

Tadekinig-α is a recombinant human IL-18-binding protein (rhIL-18BP). So far, there have been many results that can prove a favorable response toward tadekinig-α in MAS (ClinicalTrials.gov Identifier: NCT02398435, NCT03113760, NCT03512314). In an open-label, multicenter, dose-escalating phase II clinical trial, the use of tadekinig-α led to clinical improvement in 50% of patients with AOSD, and although there are some adverse events such as upper airway infections and arthralgia, most of them were mild and resolved after drug discontinuation (182). In addition, a recent case report described that MAS associated with XIAP deficiency was successfully controlled by using tadekinig-α (183).

At present, no relevant research has been found to prove the role of tadekinig- α in COVID-19. However, it still provides a new thought to prevent the occurrence of MAS and ARDS in patients with COVID-19 and improve the survival rate of patients.

#### 3.5.4 IFN-γ

Emapalumab is a fully human monoclonal anti-IFN-γ antibody (184). In November 2018, emapalumab (Gamifant) obtained an FDA indication for the treatment of children and adults with relapsed/refractory (not newly diagnosed) HLH. Recently, Michael Ryan et al. report a case of CAR T therapy-associated MAS/HLH that was successfully treated with emapalumab in combination with anakinra and corticosteroids (185). After using Anakinra, tocilizumab, and dexamethasone, the patient’s clinical symptoms did not improve. However, after the addition of emapalumab, the patient became much more stable, and achieve rapid defervescence. In an open label, single arm, phase 2 study, a total of 14 patients were enrolled, of which 13 patients achieved clinical remission (186).

Erkan Cure et al. believed that emapalumab can be life saving for cytokine storm caused by COVID-19, which is resistant to anakinra, tocilizumab, and JAK inhibitors (187). In a recent case report, this is the first case of a pediatric patient with COVID-19 associated sHLH successfully treated with emapalumab (188). The patient in this case had symptoms relapse after using etoposide and dexamethasone, so she received HCT after using emapalumab, and she has been in remission after treatment. Although this shows that the application of this drug is promising, the case report cannot fully prove its therapeutic effect. Clinical trials with a large sample size are needed to scrutinize the efficacy of emapalumab in MAS caused by COVID-19.

Moreover, like the previous drugs, the time of application of Emapalumab is also very important, because at some stages, cytokines are crucial to eliminate SARS-CoV-2, and blocking the cytokine pathway may lead to more serious consequences (189).

#### 3.5.5 JAKs pathway

Janus kinase inhibitor (JAKi) agents mainly include ruxolitinib, upadacitinib, baricitinib, tofacitinib, fedratinib, filgotinib, delgocitinib, oclacitinib and peficitinib, which act on different targets (190). Among them, ruxolitinib is the most widely used in the treatment of MAS, with a large proportion exhibiting favorable responses (191).

Compared with targeting any cytokine alone, ruxolitinib may produce more extensive anti-inflammatory activity, because these cytokines, such as IL-2, IL-6, IL-7, IL-10, IFN-γ, G-CSF, and GM-CSF, signals signal through the JAKs pathway (192). Ofer Levy et al. described a nearly fatal case of a young patient, which has been refractory to corticosteroids (CS), anakinra, tocilizumab, cyclosporine A (CSA), and etoposide, but eventually responded miraculously to salvage therapy with ruxolitinib (193). Josée-AnneJolyMSc et al. found that a therapy combining inhibition of JAK-dependent cytokines using ruxolitinib in clinically relevant doses with low doses of cytokine-specific antibodies against IFN-γ work in concert to temper the inflammatory response and control the overt activation of antigen-specific T cells, leading to a rapid and complete resolution of HLH in mice (194). Triebwasser et al. also described a patient with chronic active EBV-associated HLH who was refractory to HLH-2004 and anakinra but responded to a combination of emapalumab and ruxolitinib therapy (195).

Therefore, the potential of ruxolitinib for the treatment of severe COVID-19 has been concerned for a long time (192, 196, 197). However, in a recent randomized phase 3 trial (198) and a randomized, double-blind, placebo-controlled phase 3 trial (199), they have proved that ruxolitinib has no significance in the clinical improvement of patients and the reduction of 28-day mortality. Compared with the therapeutic effect of this drug in MAS, this is very surprising.

However, some scholars have come up with different results. After the combination with remdesivir, another JAK1/2 inhibitor, Baricitinib, can reduce recovery time and accelerate improvement in clinical status among patients with Covid-19 (200). In addition, in another randomized controlled trial, the combination of tofacitinib and glucocorticoid significantly reduced the 28-day mortality and the risk of respiratory failure in patients (201).

In summary, the treatment effect of the same treatment method in MAS alone and MAS caused by COVID-19 is different. This may be caused by different immune biological mechanisms. Some cytokines play a very important role in clearing the virus at the early stage of virus infection, before the appearance of MAS, and after the body is in a high inflammatory state, these cytokines will lead to the occurrence of MAS and ARDS (189). Therefore, the time of intervention, the way of use and the target population of different treatment methods are extremely important. A large number of clinical trials with more careful clinical design are needed to determine when and which treatment approaches is the most beneficial.

## 4 Conclusion

Although variants of SARS-CoV-2 are now becoming less virulent and transmissible, we still don’t know whether new variants will emerge and whether such variants will again pose a threat to public health. Therefore, the importance of further in-depth research on COVID-19 treatment cannot be made light of. This research aimed at MAS, which is involved in COVID-19 associated with worse disease severity and poorer prognosis. We summarized and analyzed the role of various therapeutic approaches in the two diseases. And found that the treatment effect of the same therapeutic approach is different (**Table 1**). This may be caused by different immune biological mechanisms between MAS alone and MAS caused by COVID-19. To deal this problem, further clinical trials and research are needed.

**Table 1:** MAS related therapeutic approach and its curative effect.

Therapeutic Approach and Target	Drug	Curative Effect’s Reference			
		MAS		MAS in COVID-19	
		Positive	Negative	Positive	Negative
Antiviral	Remdesivir	–	–	(69)	(70–72)
	Paxlovid	–	–	(73, 74)	–
Convalescent Plasma (CP) therapy	–	–	–	(75–79)	(82, 83)
Therapeutic Plasma Exchange (TPE)	–	(84–86)	–	(87–97)	–
Hemadsorption	–	(98, 100–103)	–	(104, 105)	(105)
Extensive immunosuppressive agent	Steroids	(106–111)	–	(114–120)	(107)
	calcineurin inhibitors (CNIs)	(123–128)	–	(129–135)	(136–138)
	Etoposide	(139, 140, 142)	–	(143–146)	–
IL-1	Anakinra	(111, 148–153)	–	(154–157)	(37)
	Canakinumab	(159, 160)	(158)	(161–165)	(166)
IL-6	Tocilizumab	(169, 172)	(170, 171)	(20, 173–176, 179)	(177)
IL-18	Tadekinig-α	(181, 182)	–	–	–
IFN-γ	Emapalumab	(184, 185)	–	(186–188)	–
JAKs pathway	Ruxolitinib	(190, 192–194)	–	(191, 195, 196)	(197, 198)
	Baricitinib	–	–	(199, 200)	–

## Author contributions

Z-H.T. and LMD supervised the study. TDC, SYC and LMD designed the structure. SYC search the paper and wrote the manuscript. TDC, SYC and DC drew the illustration. CZ analyzed references and design the table. All authors contributed to the article and approved the submitted version.
